# Supplementary material for: Diet Quality and Upper Gastrointestinal Cancers Risk: A Meta-Analysis and Critical Assessment of Evidence Quality
Source: Nutrients. 2020 Jun 23;12(6):1863. doi: 10.3390/nu12061863 (PMC7353231; doi:10.3390/nu12061863)
Supplement: Supplementary file 1 [file nutrients-12-01863-s001.zip › supplements/Box 1.docx]

| Diet quality index | Origin | Food components |
| --- | --- | --- |
| Diet Inflammatory Index (DII) | Based on pro/anti-inflammatory association of dietary components with inflammatory biomarkers, including IL-1β, IL-4, IL-6, IL-10, TNF-α | Includes 19 to 45 dietary components;  Pro-inflammatory components: Carbohydrate, Protein, total fat, Saturated fatty acids, Cholesterol, Vitamin B12, iron, Energy.  Anti-inflammatory components: Onion, Saffron, Garlic, Ginger, Fiber, Folic acid, Caffeine, and, Alcohol, MUFA PUFA, n-3 fatty acids, n-6 fatty acids, Niacin, Riboflavin, Vitamin B6, β carotene, Mg. |
| Mediterranen Diet Score (MDS) | Based on evident role of foods components in cardiovascular health | Includes 8 to 11 food components:  Foods with beneficiary effects: Cereals, vegetables, Fruits, Legumes, Nuts, Olive Oil, Fish, High ratio of PUFA+MUFA/SFA.  Foods with adverse effect: Milk and dairies, Meat and processed products, sweets, sweetened beverages, Saturated fatty acids, Cholesterol, Alcoholic beverages. |
| Healthy Eating Index (HEI) | Based on healthy eating guidelines without specific mechanism | Includes 12 to 14 food components  Foods with beneficiary effect: Fruits, Vegetables, Whole grain, beans, Dairy, Total protein in food, sea food, PUFA+MUFA/SFA≥2.5  Foods with adverse effect: Sodium, Empty calorie, refined grains, added sugar, PUFA+MUFA/SFA<1.2, alcohol. |

**Box 1**: The origin and food components of DII, MDS and HEI applied for quantifying diet quality

Abbreviations: DII, Diet Inflammatory Index; HEI, Healthy eating index; IL, Interleukin; MDS, Mediterranean Diet Score; Mg, Magnesium; MUFA, Mono Unsaturated Fatty Acids; PUFA, Poly Unsaturated Fatty Acids; SFA, Saturated Fatty Acids.
