# Supplementary material for: Diet Quality and Upper Gastrointestinal Cancers Risk: A Meta-Analysis and Critical Assessment of Evidence Quality
Source: Nutrients. 2020 Jun 23;12(6):1863. doi: 10.3390/nu12061863 (PMC7353231; doi:10.3390/nu12061863)
Supplement: Supplementary file 1 [file nutrients-12-01863-s001.zip › supplements/Finalized supplementary figures.pdf]

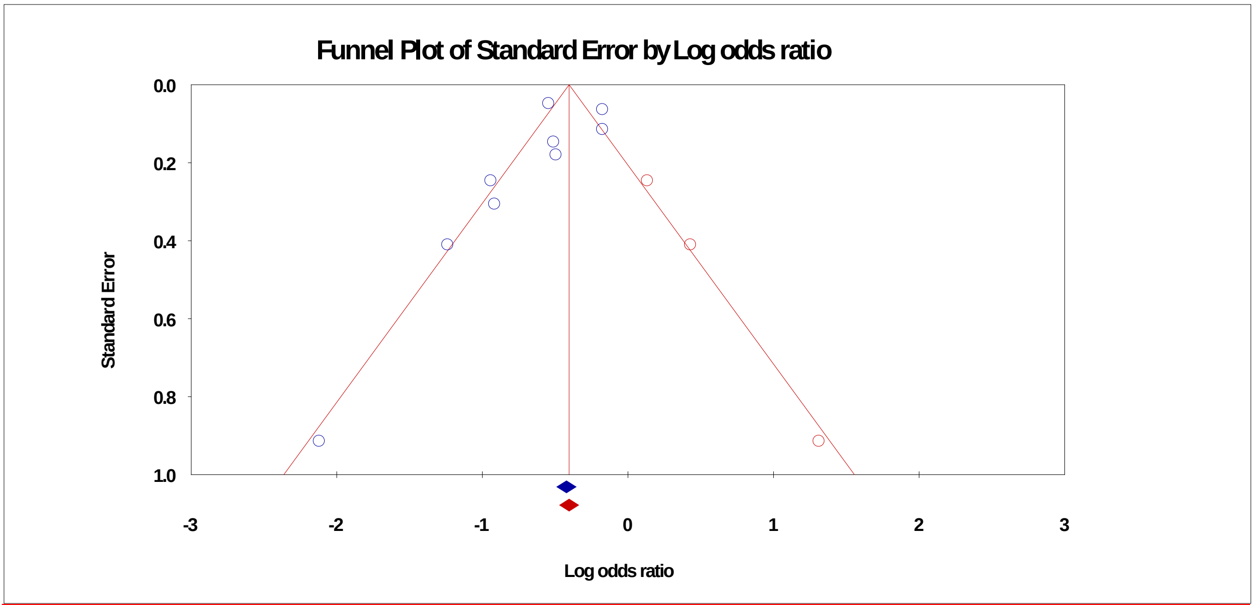

Figure S1A: Funnel plot for eligible cohort and case control studies assessing the role of high diet quality quantified by DII in risk for UGI cancers. Egger test intercept: -1.40 (95% CI: -4.16 to 1.34, p=0.26). Blue dots represent included studies. Red dots represent imputed studies by Tweede trim and fill methods. Blue diamond represents the actual pooled effect size. Red Diamond represents the adjusted pooled effect size after imputing for the missing studies (4 studies were estimated to be missed) by trim and fill adjusted values and recalculating the adjusted effect size. **Abbreviations:** DDI, Diet Inflammatory Index; UGI, Upper Gastro-Intestinal.

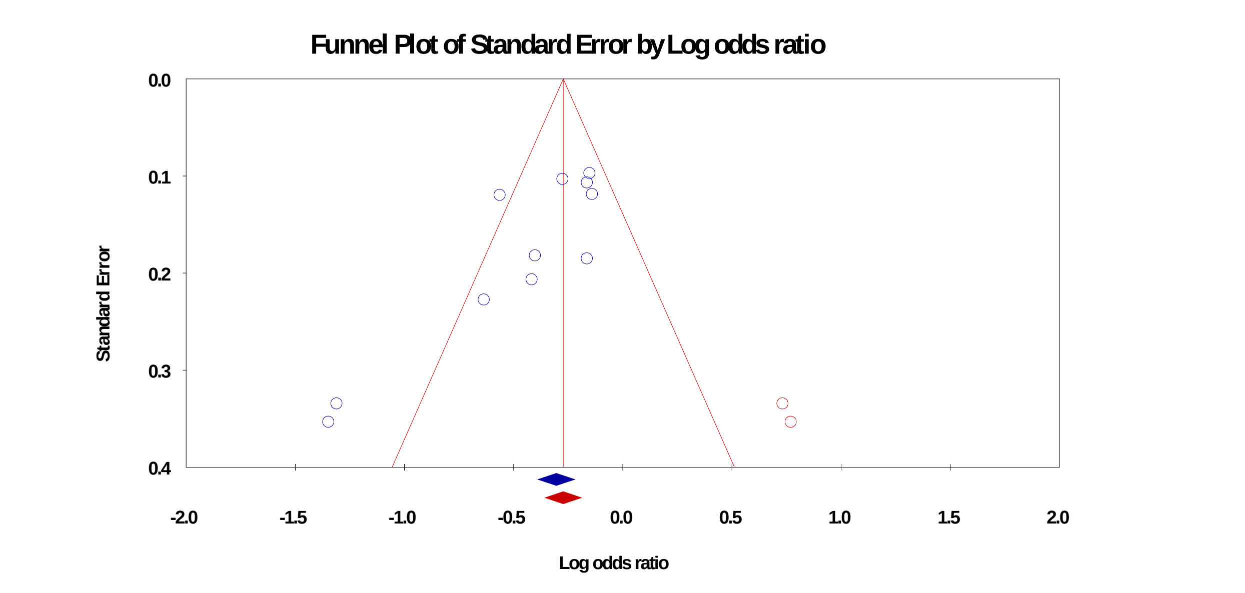

Figure S1B: Funnel plot for eligible cohort and case control studies assessing the role of diet quality quantified by MDS in risk for UGI cancers. Egger test intercept: -3.74 (95% CI:-6.47 to -1.02, P=0.01). Blue dots represent included studies. Red dots represent imputed studies by Tweede trim and fill methods. Blue diamond represents the actual effect size. Red Diamond represents the adjusted effect size after imputing for the missing studies (2 studies were estimated to be missed) by trim and fill adjusted values and recalculating the adjusted effect size. A**bbreviations:** MDS, Meditarienian Diet Index; UGI, Upper Gastro-Intestinal.

Study or Subgroup	log[Odds Ratio]	SE	Weight	Odds Ratio IV, Random, 95% CI
**1.1.1 Cohort studies**				
Agudo DII 2018	-0.5108	0.1468	46.4%	0.60 [0.45, 0.80]
Boden DII 2019	-0.1744	0.1154	53.6%	0.84 [0.67, 1.05]
**Subtotal (95% CI)**			**100.0%**	**0.72 [0.52, 1.00]**
Heterogeneity: Tau² = 0.04; Chi² = 3.25, df = 1 (P = 0.07); I² = 69%				
Test for overall effect: Z = 1.97 (P = 0.05)				
**1.1.2 Case control**				
Shivappa DII 2016	-2.1203	0.9142	2.0%	0.12 [0.02, 0.72]
Vahid DII 2018	-1.2379	0.4094	7.7%	0.29 [0.13, 0.65]
Tang DII 2018	-0.9416	0.2477	13.7%	0.39 [0.24, 0.63]
Shivappa DII 2015	-0.9163	0.305	11.1%	0.40 [0.22, 0.73]
Abe DII 2018	-0.5447	0.0502	24.3%	0.58 [0.53, 0.64]
Lee DII 2017	-0.4943	0.1784	17.5%	0.61 [0.43, 0.87]
Shivappa 1 DII 2016	-0.1744	0.0647	23.7%	0.84 [0.74, 0.95]
**Subtotal (95% CI)**			**100.0%**	**0.53 [0.41, 0.70]**
Heterogeneity: Tau² = 0.07; Chi² = 35.01, df = 6 (P < 0.00001); I² = 83%				
Test for overall effect: Z = 4.62 (P < 0.00001)				
**1.1.3 Europe**				
Shivappa DII 2015	-0.9163	0.305	10.8%	0.40 [0.22, 0.73]
Agudo DII 2018	-0.5108	0.1468	24.6%	0.60 [0.45, 0.80]
Shivappa 1 DII 2016	-0.1744	0.0647	35.8%	0.84 [0.74, 0.95]
Boden DII 2019	-0.1744	0.1154	28.9%	0.84 [0.67, 1.05]
**Subtotal (95% CI)**			**100.0%**	**0.71 [0.57, 0.90]**
Heterogeneity: Tau² = 0.03; Chi² = 9.67, df = 3 (P = 0.02); I² = 69%				
Test for overall effect: Z = 2.88 (P = 0.004)				
**1.1.4 Asia**				
Shivappa DII 2016	-2.1203	0.9142	2.2%	0.12 [0.02, 0.72]
Vahid DII 2018	-1.2379	0.4094	9.3%	0.29 [0.13, 0.65]
Tang DII 2018	-0.9416	0.2477	18.8%	0.39 [0.24, 0.63]
Abe DII 2018	-0.5447	0.0502	43.5%	0.58 [0.53, 0.64]
Lee DII 2017	-0.4943	0.1784	26.3%	0.61 [0.43, 0.87]
**Subtotal (95% CI)**			**100.0%**	**0.49 [0.38, 0.65]**
Heterogeneity: Tau² = 0.04; Chi² = 8.22, df = 4 (P = 0.08); I² = 51%				
Test for overall effect: Z = 5.06 (P < 0.00001)				
**1.1.5 Men**				
Shivappa Men 2016	-0.1625	0.0847	54.3%	0.85 [0.72, 1.00]
Boden Men 2019	0.3075	0.162	45.7%	1.36 [0.99, 1.87]
**Subtotal (95% CI)**			**100.0%**	**1.05 [0.67, 1.67]**
Heterogeneity: Tau² = 0.09; Chi² = 6.61, df = 1 (P = 0.01); I² = 85%				
Test for overall effect: Z = 0.22 (P = 0.82)				
**1.1.6 Women**				
Shivappa DII women 2016	-0.2231	0.0905	65.9%	0.80 [0.67, 0.96]
Boden Women 2019	0.0296	0.1687	34.1%	1.03 [0.74, 1.43]
**Subtotal (95% CI)**			**100.0%**	**0.87 [0.69, 1.10]**
Heterogeneity: Tau² = 0.01; Chi² = 1.74, df = 1 (P = 0.19); I² = 43%				
Test for overall effect: Z = 1.14 (P = 0.25)				
**1.1.7 Esophagus cancer**				
Tang DII 2018	-0.9416	0.2477	0.0%	0.39 [0.24, 0.63]
Shivappa DII 2015	-0.9163	0.305	18.1%	0.40 [0.22, 0.73]
Abe DII 2018	-0.5447	0.0502	41.4%	0.58 [0.53, 0.64]
Shivappa 1 DII 2016	-0.1744	0.0647	40.5%	0.84 [0.74, 0.95]
**Subtotal (95% CI)**			**100.0%**	**0.63 [0.45, 0.88]**
Heterogeneity: Tau² = 0.07; Chi² = 23.20, df = 2 (P < 0.00001); I² = 91%				
Test for overall effect: Z = 2.71 (P = 0.007)				
**1.1.8 Gastric cancer**				
Vahid DII 2018	-1.2379	0.4094	10.3%	0.29 [0.13, 0.65]
Agudo DII 2018	-0.5108	0.1468	29.9%	0.60 [0.45, 0.80]
Lee DII 2017	-0.4943	0.1784	26.4%	0.61 [0.43, 0.87]
Boden DII 2019	-0.1744	0.1154	33.4%	0.84 [0.67, 1.05]
**Subtotal (95% CI)**			**100.0%**	**0.63 [0.46, 0.84]**
Heterogeneity: Tau² = 0.06; Chi² = 8.77, df = 3 (P = 0.03); I² = 66%				
Test for overall effect: Z = 3.09 (P = 0.002)				
**1.1.9 Overall**				
Shivappa DII 2016	-2.1203	0.9142	1.2%	0.12 [0.02, 0.72]
Vahid DII 2018	-1.2379	0.4094	4.9%	0.29 [0.13, 0.65]
Tang DII 2018	-0.9416	0.2477	9.2%	0.39 [0.24, 0.63]
Shivappa DII 2015	-0.9163	0.305	7.3%	0.40 [0.22, 0.73]
Abe DII 2018	-0.5447	0.0502	18.2%	0.58 [0.53, 0.64]
Agudo DII 2018	-0.5108	0.1468	13.8%	0.60 [0.45, 0.80]
Lee DII 2017	-0.4943	0.1784	12.3%	0.61 [0.43, 0.87]
Shivappa 1 DII 2016	-0.1744	0.0647	17.7%	0.84 [0.74, 0.95]
Boden DII 2019	-0.1744	0.1154	15.4%	0.84 [0.67, 1.05]
**Subtotal (95% CI)**			**100.0%**	**0.59 [0.48, 0.72]**
Heterogeneity: Tau² = 0.06; Chi² = 40.20, df = 8 (P < 0.00001); I² = 80%				
Test for overall effect: Z = 5.02 (P < 0.00001)				

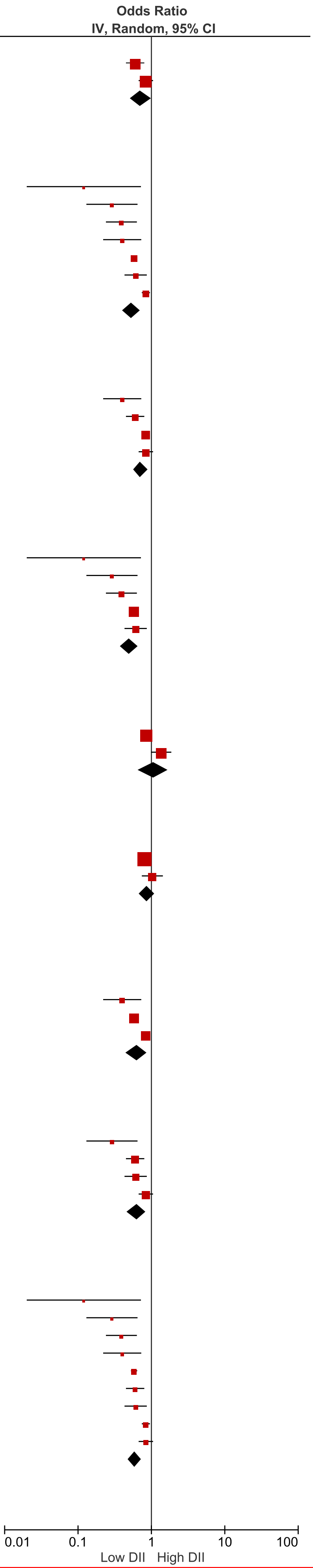

Figure S2A: Forest plot of included studies reporting the OR for highest diet quality compared to the lowest, measured by DII with respect to UGI cancers. Weights are reported by Random-effect analysis. **Abbreviations**: DII, Diet Inflammatory Index; OR, Odds Ratio; UGI, Upper Gastro-Intestinal.

Study or Subgroup	log[Odds Ratio]	SE	Weight	Odds Ratio IV, Random, 95% CI
**2.1.1 Cohort studies**				
Buckland MDS 2010	-0.4005	0.1809	7.9%	0.67 [0.47, 0.96]
Shulpen MDS 2019	-0.2744	0.1039	23.9%	0.76 [0.62, 0.93]
Boden MDS 2019	-0.1625	0.1064	22.8%	0.85 [0.69, 1.05]
Li MDS 2013	-0.1508	0.0978	27.0%	0.86 [0.71, 1.04]
Buckland MDS 2015	-0.1393	0.1183	18.4%	0.87 [0.69, 1.10]
**Subtotal (95% CI)**			**100.0%**	**0.82 [0.74, 0.90]**
Heterogeneity: Tau² = 0.00; Chi² = 2.38, df = 4 (P = 0.67); I² = 0%				
Test for overall effect: Z = 3.95 (P < 0.0001)				
**2.1.2 Case control studies**				
Bosetti MDS 2003	-1.3471	0.3537	11.1%	0.26 [0.13, 0.52]
Stojanovic MDS 2017	-1.3093	0.3351	11.8%	0.27 [0.14, 0.52]
Casteli MDS 2017	-0.6349	0.2265	16.9%	0.53 [0.34, 0.83]
Praud MDS 2013	-0.5621	0.1206	23.0%	0.57 [0.45, 0.72]
Turati MDS 2010	-0.4155	0.2069	18.0%	0.66 [0.44, 0.99]
Wang MDS 2016	-0.1625	0.1863	19.2%	0.85 [0.59, 1.22]
**Subtotal (95% CI)**			**100.0%**	**0.52 [0.39, 0.71]**
Heterogeneity: Tau² = 0.09; Chi² = 15.10, df = 5 (P = 0.010); I² = 67%				
Test for overall effect: Z = 4.21 (P < 0.0001)				
**2.1.3 US/Canada**				
Li MDS 2013	-0.1508	0.0978	100.0%	0.86 [0.71, 1.04]
**Subtotal (95% CI)**			**100.0%**	**0.86 [0.71, 1.04]**
Heterogeneity: Not applicable				
Test for overall effect: Z = 1.54 (P = 0.12)				
**2.1.4 Europe**				
Casteli MDS 2017	-0.6349	0.2265	7.7%	0.53 [0.34, 0.83]
Praud MDS 2013	-0.5621	0.1206	17.0%	0.57 [0.45, 0.72]
Turati MDS 2010	-0.4155	0.2069	8.8%	0.66 [0.44, 0.99]
Buckland MDS 2010	-0.4005	0.1809	10.6%	0.67 [0.47, 0.96]
Shulpen MDS 2019	-0.2744	0.1039	19.4%	0.76 [0.62, 0.93]
Boden MDS 2019	-0.1625	0.1064	19.1%	0.85 [0.69, 1.05]
Buckland MDS 2015	-0.1393	0.1183	17.3%	0.87 [0.69, 1.10]
**Subtotal (95% CI)**			**100.0%**	**0.72 [0.62, 0.83]**
Heterogeneity: Tau² = 0.02; Chi² = 11.06, df = 6 (P = 0.09); I² = 46%				
Test for overall effect: Z = 4.63 (P < 0.00001)				
**2.1.5 Asia**				
Wang MDS 2016	-0.1625	0.1863	100.0%	0.85 [0.59, 1.22]
**Subtotal (95% CI)**			**100.0%**	**0.85 [0.59, 1.22]**
Heterogeneity: Not applicable				
Test for overall effect: Z = 0.87 (P = 0.38)				
**2.1.6 Women**				
Bosetti MDS 2003	-1.3471	0.3537	12.5%	0.26 [0.13, 0.52]
Casteli MDS 2017	-0.6349	0.2265	19.9%	0.53 [0.34, 0.83]
Turati MDS 2010	-0.4155	0.2069	21.4%	0.66 [0.44, 0.99]
Scholpen MDS W3	-0.3425	0.4933	7.8%	0.71 [0.27, 1.87]
Scholpen MDS W4	-0.1625	0.4827	8.1%	0.85 [0.33, 2.19]
Scholpen MED W1	-0.1625	0.2129	20.9%	0.85 [0.56, 1.29]
Scholpen MDS W2	0.1222	0.4368	9.4%	1.13 [0.48, 2.66]
**Subtotal (95% CI)**			**100.0%**	**0.64 [0.47, 0.87]**
Heterogeneity: Tau² = 0.07; Chi² = 11.06, df = 6 (P = 0.09); I² = 46%				
Test for overall effect: Z = 2.83 (P = 0.005)				
**2.1.7 Men**				
Bosetti MDS 2003	-1.3471	0.3537	11.7%	0.26 [0.13, 0.52]
Schulpen MDS MEN2	-1.0498	0.4675	8.6%	0.35 [0.14, 0.88]
Scholpen MDS MEN 4	-0.734	0.3128	13.1%	0.48 [0.26, 0.89]
Casteli MDS 2017	-0.6349	0.2265	16.4%	0.53 [0.34, 0.83]
Scholpen MDS MEN3	-0.4308	0.1876	17.9%	0.65 [0.45, 0.94]
Turati MDS 2010	-0.4155	0.2069	17.1%	0.66 [0.44, 0.99]
Schulpen MDS MEN1	0.2852	0.253	15.3%	1.33 [0.81, 2.18]
**Subtotal (95% CI)**			**100.0%**	**0.58 [0.41, 0.81]**
Heterogeneity: Tau² = 0.13; Chi² = 17.94, df = 6 (P = 0.006); I² = 67%				
Test for overall effect: Z = 3.18 (P = 0.001)				
**2.1.8 Esophagus cacner**				
Bosetti MDS 2003	-1.3471	0.3537	18.0%	0.26 [0.13, 0.52]
Shulpen MDS 2019	-0.2744	0.1039	40.7%	0.76 [0.62, 0.93]
Li MDS 2013	-0.1508	0.0978	41.3%	0.86 [0.71, 1.04]
**Subtotal (95% CI)**			**100.0%**	**0.66 [0.45, 0.96]**
Heterogeneity: Tau² = 0.08; Chi² = 10.70, df = 2 (P = 0.005); I² = 81%				
Test for overall effect: Z = 2.17 (P = 0.03)				
**2.1.9 Gastric cancer**				
Praud MDS 2013	-0.5621	0.1206	35.6%	0.57 [0.45, 0.72]
Buckland MDS 2010	-0.4005	0.1809	26.3%	0.67 [0.47, 0.96]
Boden MDS 2019	-0.1625	0.1064	38.0%	0.85 [0.69, 1.05]
**Subtotal (95% CI)**			**100.0%**	**0.69 [0.53, 0.90]**
Heterogeneity: Tau² = 0.04; Chi² = 6.28, df = 2 (P = 0.04); I² = 68%				
Test for overall effect: Z = 2.71 (P = 0.007)				
**2.1.10 Overall**				
Bosetti MDS 2003	-1.3471	0.3537	4.0%	0.26 [0.13, 0.52]
Stojanovic MDS 2017	-1.3093	0.3351	4.4%	0.27 [0.14, 0.52]
Casteli MDS 2017	-0.6349	0.2265	7.1%	0.53 [0.34, 0.83]
Praud MDS 2013	-0.5621	0.1206	11.4%	0.57 [0.45, 0.72]
Turati MDS 2010	-0.4155	0.2069	7.8%	0.66 [0.44, 0.99]
Buckland MDS 2010	-0.4005	0.1809	8.8%	0.67 [0.47, 0.96]
Shulpen MDS 2019	-0.2744	0.1039	12.1%	0.76 [0.62, 0.93]
Boden MDS 2019	-0.1625	0.1064	12.0%	0.85 [0.69, 1.05]
Wang MDS 2016	-0.1625	0.1863	8.5%	0.85 [0.59, 1.22]
Li MDS 2013	-0.1508	0.0978	12.4%	0.86 [0.71, 1.04]
Buckland MDS 2015	-0.1393	0.1183	11.5%	0.87 [0.69, 1.10]
**Subtotal (95% CI)**			**100.0%**	**0.68 [0.58, 0.80]**
Heterogeneity: Tau² = 0.05; Chi² = 31.81, df = 10 (P = 0.0004); I² = 69%				
Test for overall effect: Z = 4.68 (P < 0.00001)				

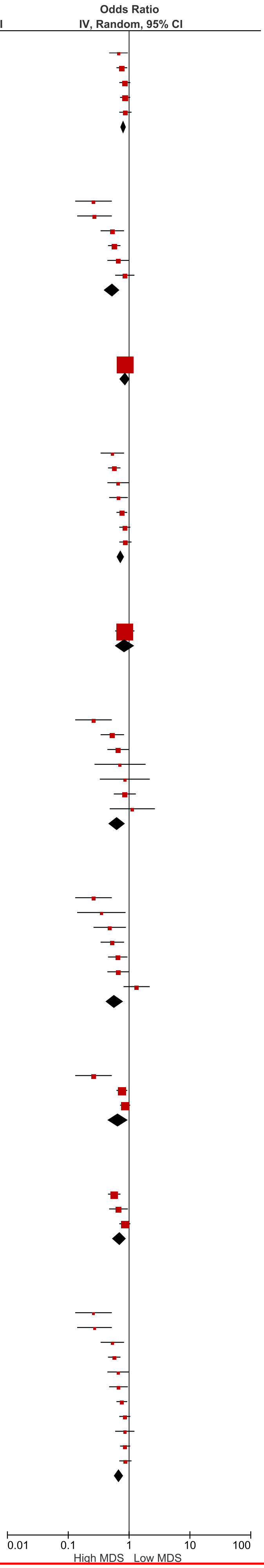

Figure S2B: Forest plot of included studies reporting OR for highest diet quality compared to the lowest, measured by MDS with respect to UGI cancers. Weights are reported by Random-effect analysis. **Abbreviations**: MDS, Mediterranean Diet Score; OR, Odds Ratio; UGI, Upper Gastro-Intestinal.
