# Supplementary material for: Diet Quality and Upper Gastrointestinal Cancers Risk: A Meta-Analysis and Critical Assessment of Evidence Quality
Source: Nutrients. 2020 Jun 23;12(6):1863. doi: 10.3390/nu12061863 (PMC7353231; doi:10.3390/nu12061863)
Supplement: Supplementary file 1 [file nutrients-12-01863-s001.zip › supplements/Table S1.docx]

**Table S1.** Search strategy for relevant literature on diet quality measured by dietary indices and risk for UGI cancers in electronic bibliographic databases.

| Database | PubMed (n=128) | EMBASE (n=33) | | Web of Sciences (n=230), Cochrane (n=90) |
| --- | --- | --- | --- | --- |
| Date | 01/02/2020 | 01/02/2020 | | 01/02/2020 |
| Strategy | #1 AND #2 | #1 AND #2 | | #1 AND #2 |
| #1 | ("Healthy Diet"[Mesh] OR Healthy Eating Index [tiab] OR Mediterranean diet [tiab] OR Inflammatory Diet Index [tiab] OR inflammatory diet [tiab] OR dietary score [tiab]) | ('healthy diet':ab,ti OR 'healthy eating index':ab,ti OR 'mediterranean diet':ab,ti OR 'inflammatory diet index':ab,ti OR 'dietary score':ab,ti) | (Healthy Diet OR Healthy Eating Index OR Mediterranean diet OR Inflammatory Diet Index OR dietary score) | |
| #2 | ("Gastrointestinal Neoplasms"[Mesh] OR "Esophageal Neoplasms"[Mesh] OR "Stomach Neoplasms"[Mesh] OR Gastric Cancer [tiab] OR Esophageal Cancer [tiab]) | ('gastrointestinal neoplasms':ab,ti OR 'esophageal neoplasms':ab,ti OR 'stomach neoplasms':ab,ti OR 'gastric cancer':ab,ti) | (Gastrointestinal Neoplasm OR Esophageal Neoplasm OR Stomach Neoplasms OR Gastric Cancer) | |

**Abbreviations:** UGI, Upper Gastro-Intestinal
